# Non-Covalent Functionalization of Carbon Nanotubes for Electrochemical Biosensor Development

**DOI:** 10.3390/s19020392

**Published:** 2019-01-18

**Authors:** Yan Zhou, Yi Fang, Ramaraja P. Ramasamy

**Affiliations:** 1 Department of Chemistry, University of Georgia, Athens, GA 30602, USA; yz1990@uga.edu; 2 Nano Electrochemistry Laboratory, School of Chemical, Materials and Biomedical Engineering, University of Georgia, Athens, GA 30602, USA; fangyi@uga.edu

**Keywords:** carbon nanotubes, bio-functionalization, non-covalent functionalization, enzyme immobilization, electrochemical biosensors, aromatic molecules, conjugated polymers

## Abstract

Carbon nanotubes (CNTs) have been widely studied and used for the construction of electrochemical biosensors owing to their small size, cylindrical shape, large surface-to-volume ratio, high conductivity and good biocompatibility. In electrochemical biosensors, CNTs serve a dual purpose: they act as immobilization support for biomolecules as well as provide the necessary electrical conductivity for electrochemical transduction. The ability of a recognition molecule to detect the analyte is highly dependent on the type of immobilization used for the attachment of the biomolecule to the CNT surface, a process also known as biofunctionalization. A variety of biofunctionalization methods have been studied and reported including physical adsorption, covalent cross-linking, polymer encapsulation etc. Each method carries its own advantages and limitations. In this review we provide a comprehensive review of non-covalent functionalization of carbon nanotubes with a variety of biomolecules for the development of electrochemical biosensors. This method of immobilization is increasingly being used in bioelectrode development using enzymes for biosensor and biofuel cell applications.

## 1. Introduction

**Figure 1:**
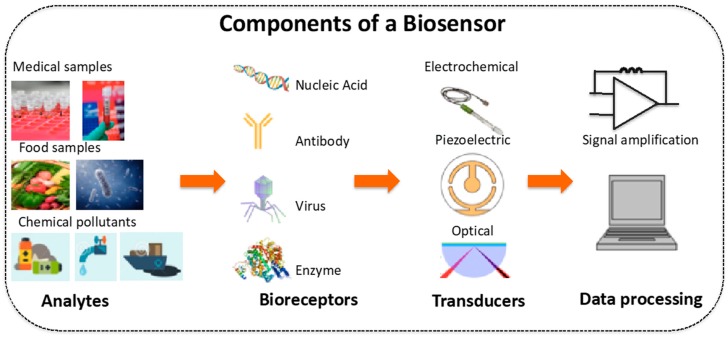
Components of a typical biosensor.

Biosensors are devices incorporating biological elements with unique binding specificities towards target analytes. A typical biosensor constitutes of three components as shown in Figure 1, a bio-receptor also called as the recognition molecule (enzyme, protein, antibody, DNA, virus, etc.), a transducer element and a signal processor [1]. The interaction between the analyte (target) and the recognition molecule is captured as a signal by the transducer which is then used for detection through signal transduction. The signal transduction could be electrochemical, magnetic, optical, colorimetric or gravimetric. Biosensors could be used in food safety, drug delivery, medical diagnosis and health care, environmental monitoring and military applications [2,3,4]. Among the above, healthcare continues to be the most important application for biosensors. Since the development of the first biosensor for oxygen detection in 1956, many other types of biosensors including the ground breaking glucose biosensor for clinic diabetes measurement have been developed [4].

Electrochemical biosensors such as the glucose biosensors possess many advantages over other types of biosensors in terms of sensitivity, cost and real time sampling capability. They can be subclassified into amperometric (current change), potentiometric (potential change) and impedimetric (impedance change) biosensors based on their transduction mechanisms [1]. Nanomaterials such as gold nanoparticles, metal oxide nanoparticles, carbon nanotubes and quantum dots, etc. are used as immobilization support for recognition molecules in electrochemical biosensors. They also aid in enhancing the signal sensitivity during detection [5,6,7,8,9]. Compared to other nanomaterials, carbon nanotubes (CNTs) have been recognized as one of the most important material for electrochemical transduction in biosensors due to their fast electron transfer capability, high surface area for signal amplification and high chemical stability [10]. They possess distinct electronic properties resulting from their unique structure [11,12,13] and are well suited for the transduction of electrical signals generated upon recognition of a target [14]. CNTs have been studied extensively for their mechanical, thermal and electronic properties and as well as for their interactions with molecular, ionic or macromolecular chemical species. The chemical properties of CNTs could be changed permanently or reversibly through various chemical modifications of their surfaces to impart new functionalities. In this review, a non-covalent functionalization of CNTs is explored and discussed in detail. The purpose of non-covalent functionalization is to enhance the bio-affinity of CNTs as well as to establish effective electrical communication between the bioreceptor and the transducer in biosensor platforms, that will contribute to signal amplification and transduction. Different functionalization strategies for non-covalent functionalization using aromatic compounds, polymers and amphiphilic molecules are discussed. Further, the application of CNT-based electrochemical biosensors using versatile bio-recognition elements such as DNA, antibodies, enzymes, etc. are also discussed in depth [15,16].

**Figure 2:**
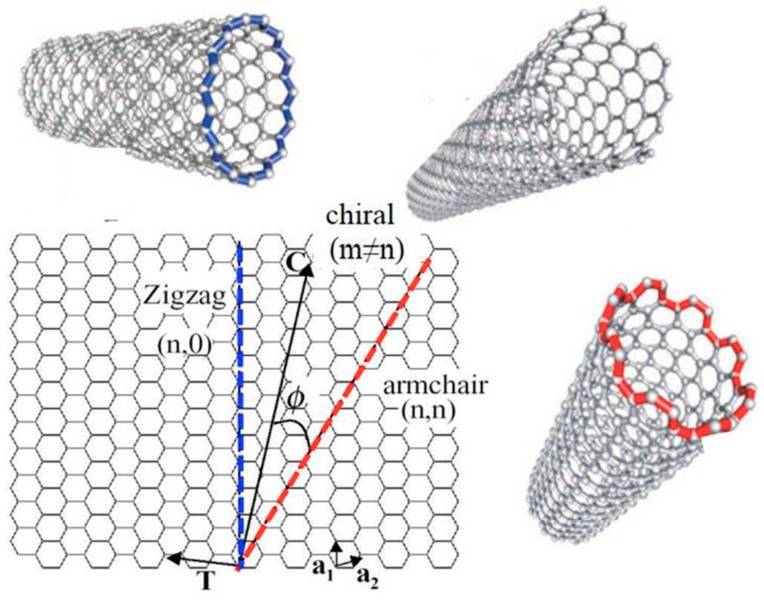
Diagram showing the various possible rolling directions of graphene that results in single wall carbon nanotubes with different chiralities [14].

Carbon nanotubes are made of sp^2^ carbons arranged as hollow cylindrical tubes, with diameters ranging from 1 to 100 nm. Two forms of carbon nanotubes are commonly available, single wall carbon nanotubes (SWCNTs) and multiwall carbon nanotubes (MWCNTs). SWCNT is the rolled form of graphene sheet, while MWCNT consists of multiple layers of concentric single walled graphene cylinders held together by Van der Waals forces with an interlayer spacing of 3.4 Å [17]. CNTs consist of hexagonal rings, which define their diameter, curvature and electronic properties. The type of carbon hexagonal ring arrangement in a nanotube is called chirality of CNTs. SWCNTs can be either semi-conducting or semi metallic depending on their diameter and chirality [18]. As shown in Figure 2, SWCNTs can exhibit different chiralities such as armchair, zigzag or chiral depending on its rolling direction. The chiral vector **C_h_**, **C_h_** = *n***α_1_** + *m***α_2_**, determines the chirality of a SWCNT, where *n* and *m* are integers, and **α_1_**, **α_2_** are the lattice vectors of graphene. Chirality plays an important role in determining the physical properties of SWCNTs such as their electronic conductivity. For instance, SWCNT with an armchair structure, when *n* = *m* (*n*, *n*), has no band gap and is always metallic. When *m* = 0, SWCNT takes a zig-zag chirality with either metallic or semi-conductive property. Chiral-structured SWCNT (*n* > *m* > 0) can also be metallic if *n* = 3*q* (*q* is an integer) [14,19,20,21]. In the case of MWCNTs, where graphene sheets are rolled up in concentric cylinders, that can be described by two structural models: Russian Doll model and Parchment model. In the Russian Doll model, the outer nanotube has a greater diameter than the inner nanotube. In the Parchment model, nanotube is rolled around itself as a rolled paper. MWCNT is metallic if one sheet has metallic chirality [22]. The electron transport in MWCNTs has also been observed to be comparable to that of SWCNTs since most of the current passing through is limited to the outermost layer [23,24].

Although CNTs can be regarded as the rolled-up form of graphite, substantial differences in physical and chemical properties exist between the two classes of materials [14,17,23,25,26]. The carbon atoms in a CNT are pyramidalized due to the curvature of the CNT sidewall. Curvature in the nanotube also introduces misalignment of the π-orbitals within the graphene sheet. It has been reported as the diameter of the CNT increases, both pyramidalization and π-orbital misalignment decreases, which renders lowered chemical reactivity of the carbon bond, eventually approaching planar graphite properties for large CNT diameters [17,18]. Additionally, rolling the graphene sheet increases the reactivity of the convex surface while decreasing the reactivity of the concave surface compared to the planar graphene sheet. The activity of the immobilized molecules on the exterior of the CNT is reported to be higher compared to that on the interior of the CNT [18]. Although the chemical and electronic properties of the CNTs are widely reported, the influence of chirality on biomolecule functionalization is rarely studied [18,27]. Tournus and Charlier theoretically studied the immobilization of benzene on the exterior surface of chiral and armchair SWCNTs using Discrete Fourier Transform method [28]. They found that the immobilization of benzene on SWCNT was strongest when there was minimal π-orbital misalignment (i.e., maximum diameter). This implies that the interaction between CNT and the immobilized molecule is strongly dependent on the π-orbital orientation and CNT curvature. The main purpose of this review is to present the recent advances in non-covalent functionalization of CNTs and their applications in the field of electrochemical biosensors.

## 2. Functionalization of Carbon Nanotubes

Bio-functionalization is the process of immobilizing biomolecules onto surfaces in order to impart the surfaces with specific functions such as bio-specificity and/or catalytic activity. The primary intent of bio-functionalization is to prepare the surface for a specific application such as biosensors. Strategies for immobilizing various biomolecules such as proteins, enzymes, antibodies and nucleic acids, onto CNTs have been extensively studied and widely used in numerous biosensor applications [14,29,30,31,32,33,34]. The high surface-to-volume ratio of CNTs allows high biomolecule loading per unit geometric area that aids in high signal amplification. Herein, we classify the bio-functionalization of CNTs into two categories: covalent functionalization and non-covalent functionalization.

### 2.1. Covalent Functionalization

**Figure 3:**
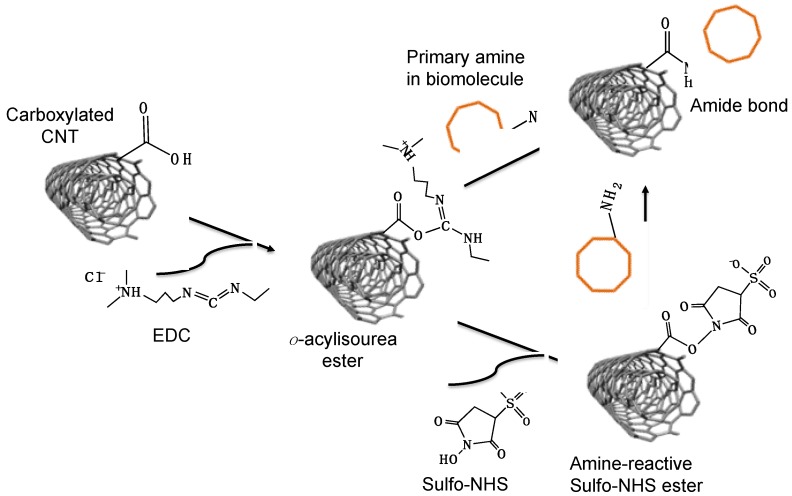
Reaction scheme for EDC and EDC-NHS based covalent crosslinking of biomolecule with carbon nanotube.

Covalent functionalization of CNT can be achieved by introducing chemical functional groups on the CNT sidewalls to result in carboxylated CNTs, amine functionalized CNTs, or sulfhydryl functionalized CNTs to mention but a few. The functional groups on the CNTs could react with complementary functional groups present in the biomolecule structure to form a covalent bond that aids in the attachment (immobilization) of the biomolecule on the CNT surface. For example, carboxylated CNTs can react with biomolecules using a carbodiimide compound, which could activate carboxyl groups on CNT for direct reaction with primary amines in biomolecules. The commonly used water-soluble carbodiimide is N-ethyl-N’-(3-dimethylaminopropyl) carbodiimide hydrochloride (EDC). EDC reacts with carboxyl groups and form an intermediate *o*-acylisourea ester, which could be easily displaced by primary amine in the biomolecule. The scheme of EDC crosslinking chemistry is shown in Figure 3. Thus, an amide bond is formed between carboxyl group and the primary amine. For example, Zhang et al. reported immobilization of horseradish peroxidase using poly-l-lysine as cross-linker and EDC as cross-linking agent [35]. N-hydroxysuccinimide (NHS) or Sulfo-NHS is often used to facilitate the coupling reaction. The EDC-NHS coupling method forms NHS ester which is more stable than the intermediate *o*-acylisourea ester, since *o*-acylisourea ester is unstable in aqueous solution.

**Figure 4:**
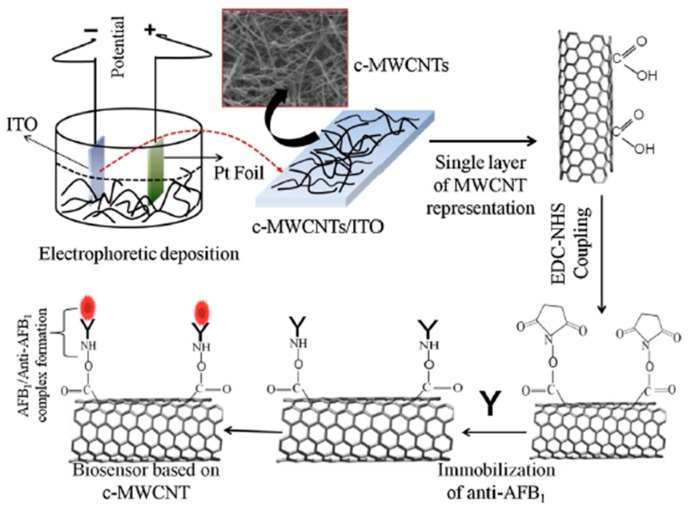
Schematic of MWCNT based biosensor for aflatoxin B1 detection [37].

Several proteins have been studied using this method of immobilization onto carboxylated CNT surface. Zeng et al. covalently functionalized CNT with poly(amidoamine) (PAMAM) dendrimer to increase the solubility of CNT in water. The resulting CNT was further used for immobilization of glucose oxidase and horseradish peroxidase (HRP) [36]. Singh et al. developed an electrochemical immunosensor based on a MWCNT deposited Indium-Tin Oxide (ITO) electrode. Monoclonal aflatoxin B1 antibody was immobilized onto electrode for detection of aflatoxin-B1. The proposed immunosensor displayed high sensitivity of 95.2 μA·ng^−1^·mL·cm^−2^ with improved detection limit of 0.08 ng·mL^−1^. The low value of association constant (0.0915 ng·mL^−1^) strongly indicates the high affinity of immunoelectrode towards aflatoxin (Figure 4) [37]. Table 1 summarizes the above examples of covalently functionalized CNT for biosensor applications.

Carboxylated CNTs can also be further modified to achieve other functionalities. Amine functionalized CNTs can react with amine groups on biomolecules such as proteins, enzymes, or antibodies. Amine-active linking agents typically include glutaraldehyde, active ester, or epoxy. Glutaraldehyde (GA), an amine reactive homo-bifunctional crosslinker, is commonly used in biochemistry applications. An imide linkage is formed upon glutaraldehyde interacting with amine containing biomolecules [40]. Viswanathan et al. developed an electrochemical immunosensor for detection of carcinoembryonic antigen (CEA) in saliva and serum. The bioreceptor monoclonal anti-CEA antibodies were covalently immobilized onto polyethyleneimine wrapped MWCNT sidewalls using 2% glutaraldehyde [38]. Similar strategy was performed on covalent immobilization of glucose oxidase onto gelatin-MWCNT through glutaraldehyde chemistry [39].

Other methods have also been developed to covalently immobilize biomolecules onto CNT surface without crosslinkers. The copper(I)-catalyzed azide–alkyne cycloaddition (CuAAC) “click” reaction could link alkyne-derivatized CNTs to an azido-derivatives, such as β-cyclodextrin derivative [41]. Dinesh et al. and Lamanna et al. have carefully reviewed the preparation of multifunctionalized CNTs through double/triple covalent functionalization [42,43].

Though covalent functionalization enables a strong interaction between the biomolecule and the CNTs, specific experimental conditions are required for covalent functionalization of carbon nanotubes in order to alter the surface functionality of carbon nanotubes. It may also lead to non-homogeneous substitution of multiple bonds per biomolecule that could reduce its activity. Moreover, the modification of CNT surface by covalent attachment could alter the intrinsic properties, such as change in hybridization from sp^2^ to sp^3^, resulting in possible loss of conjugation properties which could impact conductivity and mechanical strength [17,24,44,45].

### 2.2. Non-Covalent Functionalization

Non-covalent functionalization is quite attractive for biomolecule immobilization on CNTs because of its ability to retain the intrinsic properties of CNT when imparting new functionality. Non-covalent methods could help preserve the conformational structure of the immobilized biomolecules after immobilization on to CNT, whereas covalent approaches could affect the sp^2^ structures of carbon nanotubes, thereby negatively impacting their mechanical and electronic properties. In general, an ideal method for non-covalent functionalization of CNT should use biocompatible functionalization agents that are stable without detachment on the CNT surface under versatile environments and possess suitable functional moieties for linking to various biomolecules.

Non-covalent functionalization of CNT can be achieved by establishing π-π interactions (using aromatic compounds or polymers), electrostatic interactions, and CH-π interactions between CNTs and biomolecules [45,46,47]. Non-covalent functionalization could retain the biocompatibility of CNT without compromising its electrical or mechanical capabilities. The applications of CNTs in biosensors have been hindered for a long time due to their poor dispersion in aqueous solutions. CNTs have tendency to aggregate into bundles and ropes due to Van der Waals forces making it difficult to disperse them in many solvents. Functionalization of CNTs with high affinity molecules, such as surfactants, conjugated aromatic molecules and polymers is widely used to increase the solubility of CNTs in solvents which could enhance the adsorption of biomolecules on their surfaces. An effective functionalization method must not only introduce versatile and homogeneous surface functional groups, but also minimize its impact on the electronic properties of CNTs.

#### 2.2.1. Π-Π Interactions with Aromatic Molecules

**Figure 5:**
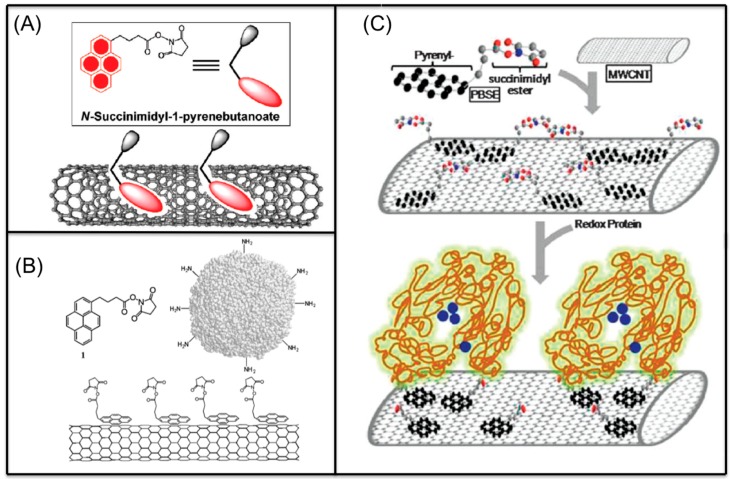
(**A**) SWCNT functionalized with pyrene-based molecular tether, 1-pyrenebutanoic acid, succinimidyl ester (PBSE) (adapted with permission from Zhao et al. Copyright (2018) American Chemical Society) [45]; (**B**) Anchored PBSE for immobilization of proteins on SWCNT (adapted with permission from Chen et al. Copyright (2018) American Chemical Society) [50]; (**C**) Multicopper oxidase immobilized on MWCNT using PBSE [55].

Molecules containing aromatic groups are capable of achieving non-covalent functionalization of CNTs. The functionalization is achieved by forming specific and directional π-π stacking interactions between aromatic molecules and the graphitic surface of nanotubes. Researchers have investigated the interactions between aromatic compounds and CNTs using field-effected transistor devices [48]. Woods et al. studied the adsorption of small aromatic molecules, benzene derivatives, on SWCNT using density-functional theory and found that the adsorption was achieved mainly through the interaction of π orbitals of the benzene ring and those of the CNT [49]. This interaction not only enhances the solubility of CNTs in the solvents, but also plays an important role in non-covalent functionalization of CNTs. Certain aromatic compounds, such as anthracene, pyrene, ferrocene and phthalocyanine derivatives, have been used for immobilization of biomolecules onto CNTs. Figure 5A shows a pyrene molecule functionalized SWCNT [45]. Table 2 displays the structures of aromatic compounds that have been explored for the development of electrochemical bioelectrodes.

Chen et al. reported for the first time, a simple and general approach to non-covalent functionalization of side-walled carbon nanotubes using 1-pyrenebutanoic acid, succinimidyl ester (PBSE). The anchored PBSE molecules on the sidewalls of SWCNT were highly resistant to desorption in aqueous solution as shown in Figure 5B, and promote the reaction between succinimidyl ester groups and primary or secondary amines of proteins. Ferritin, streptavidin and biotinyl-3,6-dioxaoctanediamine were successfully immobilized with high specificity onto SWCNT. This approach could be extended beyond biomolecules and could be utilized in various applications [27,45,50,51,52,53,54]. In our previous work, we have reported the benefit of using pyrene derivatives to non-covalently functionalize CNTs for designing enzymatic biosensors, microbial biosensors and biofuel cells [55,56,57,58,59]. We demonstrated a successful functionalization of MWCNTs with PBSE for immobilization of multicopper oxidase enzymes as catalysts on electrode surfaces for bioelectrochemical oxygen reduction reaction as shown in Figure 5C.

The PBSE being a hetero-bifunctional cross-linker, conjugates with both MWCNT and multi-copper oxidase effectively and thus facilitating the direct electron transfer between the MWCNT electrode and the enzyme for bio-electrocatalytic oxygen reduction. Giroud et al. also investigated the use of anthracene grafted pyrene derivatives to achieve direct electron transfer of laccase in oxygen reduction [64]. Multiple functionalization of SWCNTs through π-π stacking was achieved by Holzinger et al. via simple dip coating process. The functionalization was established by attaching three different pyrene derivatives onto CNTs sidewalls for the construction of glucose biosensors, including adamantane–pyrene, biotin–pyrene, and nitrilotriacetic acid (NTA)–pyrene. The prepared multifunctional electrodes were then immobilized with β-cyclodextrin modified glucose oxidase (β-CD-GO*_x_*), biotinylated glucose oxidase (B-GO*_x_*) and histidine modified glucose. The functionalization method could be applied to any kind of nanotube modified surface and nanotube fibers [61]. Wei et al. developed an impedance sensor for determination of polychlorinated biphenyl based on SWCNT/ pyrenecyclodextrin hybrid [79]. With the aid of pyrenyl group, CD could attach to the sidewalls of CNTs by means of π-π stacking. The established system is highly sensitive and selective to polychlorinated biphenyl.

**Figure 6:**
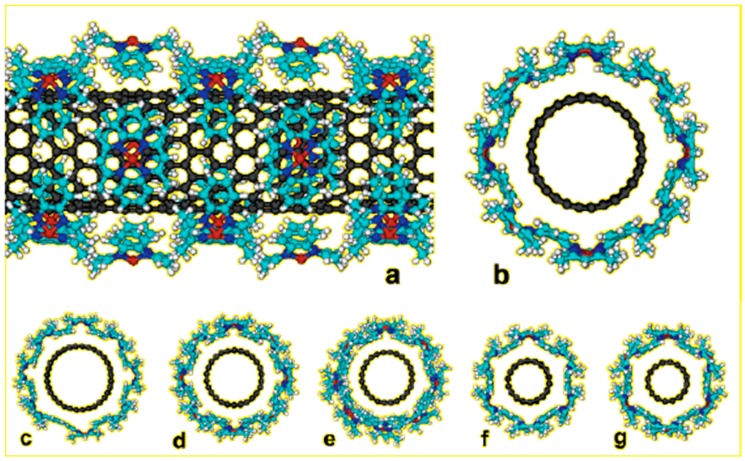
Aromatic annulene adsorbed on SWCNT walls. Exemplified for NiTMTAA and zigzag SWNTs: (**a**, **b**) (14, 0); (**c**) (16, 0); (**d**) (13, 0); (**e**) (12, 0); (**f**) (9, 0); and (**g**) (8, 0). Side view (**a**) and cross sections (**b**–**g**). Atom coloring: carbon, black (nanotube) and light-blue (complex); hydrogen, white; nitrogen [60].

Basiuk et al. studied the reversible modification of SWCNT with metal complexes, Ni (II) and Cu (II) complexes of 5,7,12,14-tetramethyldibenzo-1,4,8,11-tetraazacyclotetradeca-3,5,7,10,-12,14-hexaene (TMTAA). The aromatic annulene is distorted from the plane in the presence of four methyl substitutes interfering with the benzene ring. The resulting saddle-shaped conformation, with methyl and benzene groups turned away from MH_4_ coordination plane, resulting in the suitable curvature match of small diameter SWCNT sidewalls as shown in Figure 6. The complexes allow reversible attachment of modifying moieties. It interacts strongly with the nanotube sidewalls due to π-π interaction and remains stable in aqueous solutions. The unique feature of TMTAA makes it a suitable candidate as tethering agent for biomolecule immobilization in biosensor applications [60]. Ferrocene functionalized CNT was also studied and electrochemical systems for biosensor applications [80,81,82]. Yang et al. first reported the preparation of non-covalent nanohybrid of ferrocene functionalized SWCNT with enhanced electrochemical properties [62]. They have hypothesized that ferrocene could be immobilized on the SWCNT surface through π-π stacking and Van der Waals interactions between ferrocene molecules. The hybrid Fc/SMCNT showed enhanced catalytic property in hydrogen peroxide reduction, demonstrating its potential as an electrode material for biosensing applications.

Huang et al. have used the ferrocene non-covalent functionalized SWCNT for L-glutamate detection [63]. The functionalized ferrocene/SWCNTs demonstrated high stability not only in water but also in organic solvents, such as acetone and ethanol. Electrodes fabricated with ferrocene/SWCNT exhibited high catalytic activity and sensitivity for the detection of L-glutamate (1 μM). Recently, Cluff et. al. demonstrated that ferrocene could be adsorbed onto carbon nanotube surfaces in the absence of solvent at room temperature. The interaction between ferrocene and carbon nanotube is based on Van der Waals interaction [83].

Anthracene derivatives can also be used as linking agents for non-covalent immobilization. Anthracene molecule has been substituted by different groups with various electrophilic capability to form anthrarobin, 9,10-dibromoanthracene, 9,10-anthracenedicarbonitrile and 9-anthracene-methanol. Zhang et al. studied the interaction between anthracene derivatives and SWCNTs using fluorescence and UV spectroscopy [65]. The author hypothesized that the π-π interaction between SWCNTs and aromatic adsorbate is accompanied by an electron donor-acceptor charge transfer interaction. Thus, the adsorption effectiveness of anthracene derivatives to SMCNTs is determined by its electron affinity: -CN > -Br > -CH_2_OH > -H > -OH. Anthracene-modified multi-walled carbon nanotubes as direct electron transfer scaffolds for enzymatic oxygen reduction was reported by Meredith et al. The results revealed the increase of sp^2^ hybridized carbons, giving evidence for the attachment of anthracene on CNT surface [84]. Song et al. reported the use of anthracene–tetrathiafulvalene (TTF) derivative for non-covalent functionalization of SWCNT. The new anthracene-TTF encapsulated SWCNT composites were then used for the detection of DNA [65,85].

**Figure 7:**
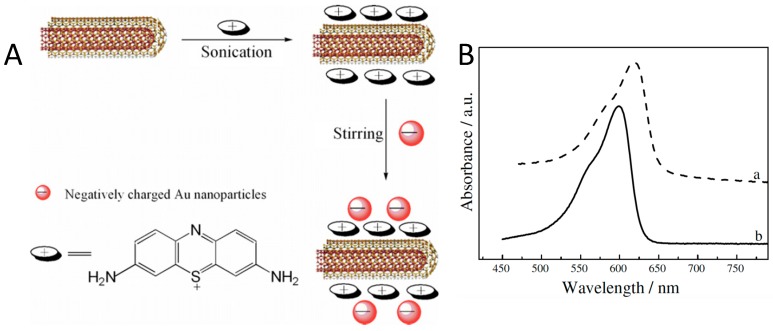
(**A**) Proposed mechanism of thionine-mediated adsorption of gold nanoparticles on MWCNTs; (**B**) UV–Vis absorption spectra of: (a) MWCNT/thionine solution and(b) aqueous thionine solution.

Thionine is a small planar molecule with phenothiazine core and two amine groups symmetrically distributed on each side. It could interact with CNT in both covalent and non-covalent manners [66,67]. In solution, thionine in protonated form (Thi^+^) could be non-covalently adsorbed onto MWCNT sidewalls through π-π stacking [66]. Wang et al. prepared thionine functionalized MWCNT/gold nanocomposites, where Thi^+^ was first adsorbed onto MWCNT through π-π interaction, followed by the subsequent binding of gold nanoparticles onto the cationic MWCNT surface (Figure 7) [67]. A red shift of the main peak in the UV-Vis spectrum (Figure 7B) suggests the strong interaction between MWCNTs and the thionine molecules.

Electrochemical investigations of the composite showed enhanced stability and electrocatalytic activity towards oxygen reduction. Furthermore, thionine was also used as mediator in biosensors, which facilitated electron transfer between biological element and electrode [68,69,70]. Hashemnia et al. investigated the use of thionine-functionalized MWCNT for immobilization of catalase (Ct) enzyme [71]. The prepared electrodes were then used for hydrogen peroxide detection with a detection limit of 8.7 μM and a sensitivity of 6.051 μA·μM^−1^·cm^−2^. 

Yan et al. introduced the non-covalent adsorption of electro-active methylene blue (MB), an aromatic electro-active dye, onto SWCNT to build a functional nanostructure. The adsorption of MB onto SWCNT revealed that the MB interacted with SWCNT through charge transfer and π-π interaction. The functional nanocomposite MB-SWCNT could be useful in many electronic applications like biosensors and photovoltaics [86]. Other organic dyes, such as Prussian blue and Congo red (CR), were also used for non-covalent functionalization of MWCNT through strong π-π interaction [72,73]. Yang et al. developed an electrochemical sensor using Congo red-functionalized MWCNT for detection of ofloxacin in human urine. The prepared soluble CR-MWCNT had high stability up to two months. The CR-functionalized CNT was also reported to form uniform and stable CNT films on different substrates which possess excellent electrochemical properties towards various substances (redox proteins, small biomolecules, hormones and so on) [87,88,89,90].

Li et al. reported a facile functionalization of SWCNT with naphthalen-1-ylmethylphosphonic acid (NYPA) through π-π stacking for biosensing applications. The resulting NYPA-SWNT hybrids were observed with negative zeta potential, confirming that the negatively charged phosphonate groups are exposed on the sidewalls of SWCNT. Positively charged myoglobin was then immobilized on the phosphonate-functionalized SWCNTs through electrostatic interaction. The fabricated biosensor showed excellent bioelectrocatalytic activity towards H_2_O_2_ reduction with a detection limit of 1.5 × 10^−7^ M [74].

Mao et al. used 1,10-phenanthroline-5,6-dione (PD) to endow CNT with redox properties that could mediate the oxidation reaction of NADH. PD was adsorbed onto CNT sidewall through π-π interactions. The PD-derived redox CNT demonstrated excellent electrocatalytic activity toward NADH oxidation and it was further applied for alcohol detection [75]. Zhang et al. reported a novel electrochemical method for sensitive detection of 2,4,6-trinitrotoluene (TNT) based on electrodes modified with non-covalent triphenylene (TP) functionalized MWCNT. TP molecule possesses π-conjugate structure, which interacts with CNT sidewalls through π-π interaction. TP-MWCNT was then used as a sensing platform for TNT detection with higher sensitivity and rapid response than that of pristine MWCNT [76].

**Figure 8:**
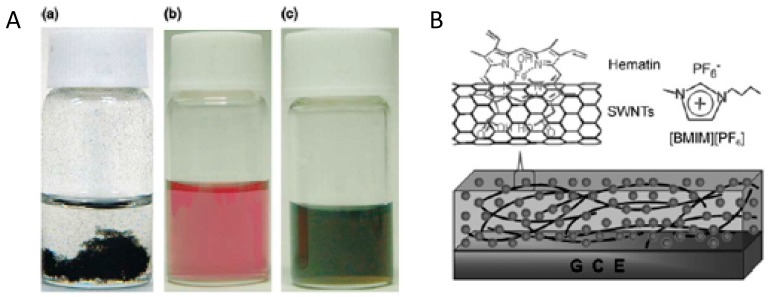
Photos of (**A)**: (a) a DMF dispersion of p-SWCNT solely, (b) a DMF solution of ZnPP and (c) a transparent DMF solution/ dispersion of p-SWCNT–ZnPP [77]. (**B**): Diagram of a porphyrin/SWCNT-[BMIM][PF_6_]-modified glassy carbon electrode (GCE) [78].

Porphyrins are important conjugated organic molecules, which could also be used for functionalization of CNT via non-covalent routes. Murakami et al. reported the preparation of porphyrin non-covalently functionalized SWCNT-porphyrin nanocomposites for the first time. Increased solubility of SWCNT-porphyrin was observed in organic solutions as shown in Figure 8A [77]. Non-covalent functionalization of CNT with porphyrins also facilitates electron transfer, leading to better performance of biosensors. Tu et al. reported the use of hydroxyferriprotoporphyrin (hematin) functionalized SWCNT in the presence of 1-butyl-3-methylimidazolium hexafluoro-phosphate ([BMIM][PF_6_]) ionic liquid for detection of trichloroacetic acid (TCA) as shown in Figure 8B. The porphyrin dissolved in ionic liquid can be self-assembled onto SWCNT by π-π interaction, leading to a direct electrochemical response. The porphyrin/SWNTs–[BMIM][PF_6_]-modified GCE showed excellent electrocatalytic activity towards the reduction of TCA, producing a highly sensitive biosensor for TCA [78].

#### 2.2.2. Π-Π Interaction with Polymers

**Figure 9:**
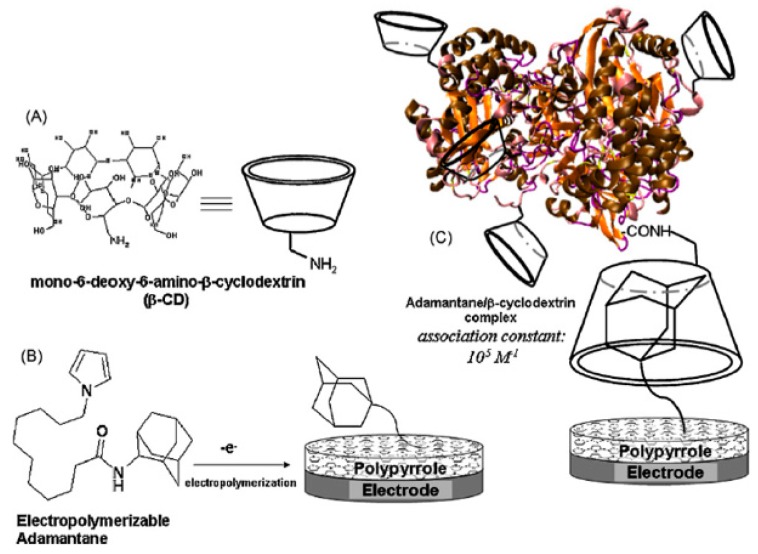
(**A**) Schematic of β-CD; (**B**) Electrogeneration of poly(adamantane-pyrrole) on the electrode surface; (**C**) β-CD-tagged GO*x* and adamantane-modified electrodes [98].

Polymers provide three-dimensional nanostructures with large electro-active area, which can also be used to non-covalently functionalize CNTs for biosensor applications. Polymers such as cellulose derivatives, polypyrroles, glycolipids, and redox polymers have been engineered for functionalization of CNTs to explore and develop its applications [91,92,93]. CNT/polypyrrole films have been well studied since the resulting complex could enhance conductivity and stability, and improve electron transfer reactions of biomolecules [94]. SWCNT/polypyrrole composite used as working electrode dramatically increases the direct electron transfer of multihemic nitrite reductase by 10-fold increase in catalytic current compared to bare glassy carbon electrode. The polymer layers also help to preserve the electroenzymatic activity of the working electrode for several months [95]. Table 3 shows the structure of polymers that have been applied in electrochemical biosensors discussed in this review. Zhu et al. reported an amperometric biosensor using electropolymerized pyrrole functionalized SWCNT with glucose oxidase entrapped in the matrix. Direct electron communication was established by non-covalent functionalized SWCNT between enzyme and electrode [96]. Electropolymerization was performed at optimal range between −0.8 V and +0.8 V in electrochemical cell containing pyrroles [97]. Holzinger et al. reported the synthesis and electropolymerization of an adamantane-pyrrole derivative as a new affinity binding polymer for construction of an amperometric glucose biosensor (Figure 9). 

The biosensor was based on interactions between polymerized adamantane and β-cyclodextrin conjugated molecules mimicking the biological avidin-biotin interaction. SWCNT was firstly functionalized with electropolymerized adamantane-pyrrole, followed by the layering with β-cyclodextrin gold nanoparticles as intermediates. Adamantane-tagged glucose oxidase was then anchored onto gold nanoparticles leading to an efficient amperometric glucose biosensor [98]. The combination of SWCNT, polymers and gold nanoparticles offer a specific surface for protein immobilization and could be applied for development of various biosensors. Ping et al. developed an amperometric biosensor for glucose detection using conjugate polymer poly[3-(3-N,N-diethylaminopropoxy) thiophene] (PDAOT). The synthesized PDAOT was employed in functionalization of SWCNT. The complex PDAOT-SWCNT is highly stable in aqueous solutions and the non-covalent functionalization did not change the nanotube structure and properties based on UV-Vis and Raman spectroscopy studies [99]. Polyaniline (PANI) has also been studied due to its extended π-conjugated system. The 3D-structure of the polymer allows the adsorption of enzymes with promoted interaction between the carboxyl groups of the enzyme and the amino groups in the polymer chain. Cesarino et al. developed a sensitive electrochemical biosensor using PANI modified-MWCNT for detection of carbamate pesticides in fruits and vegetables based on the enzymatic inhibition of acetylcholinesterase (AChE). PANI modified MWCNT generated cavities where enzymes could be properly immobilized [93]. Recently, glycoconjugate-functionalized CNTs have been shown to possess great potential for biosensor applications [53,106]. For example, glycolipids, which are amphiphilic molecules, composed of both carbohydrate polar head and a lipid hydrophobic tail, could interact with SWCNTs providing a multi-functional sensor platform. Researchers have also synthesized and investigated neutral pyrene functionalized glycolipids that could interact with CNT surface, offering opportunities for specific ligand-lectin interaction similar to glycoconjugates on the cell membrane [52]. For example, Wu et al. synthesized glycodendrimers as homogeneous bioactive coating for CNT. The pyrene tail of the glycodendrimer could bind onto SWCNT surface though π-π interaction and the promising complex can be readily adapted to biosensors for carbohydrate-binding proteins [27].

#### 2.2.3. Electrostatic Interaction with Polymers

Electrostatic interaction also plays an important role in the adsorption of biomolecules onto CNT surface. Positively charged myoglobin protein was immobilized on phosphonate functionalized SWCNTs through electrostatic interaction. The resulting electrode showed excellent bioelectrocatalytic activity toward hydrogen peroxide reduction [74]. The electrostatic interactions have also been observed between polymer-functionalized CNTs and biomolecules. Polymers coated on CNTs are either negatively charged or positively charged which could interact with opposite charged biomolecules.

**Figure 10:**
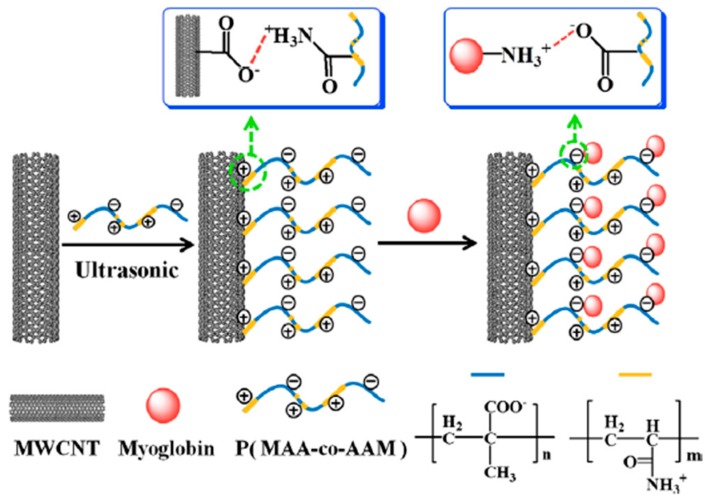
Fabrication process for the preparation of Mb-P(MAA-*co*-AAM)-MWCNT nanocomposites [100].

Sartori et al. developed a biosensor for determination of sulfite using MWCNT-gold nanoparticle (AuNP)-modified glassy-carbon electrode. The AuNPs were anchored onto the nanotubes through electrostatic interaction provided by the polymer. Poly(allylamine hydrochloride) was used to achieve electrostatic interaction between MWCNT and AuNPs [107]. Zhan et al. prepared poly(methacrylic acid-*co*-acrylamide) (P(MAA-*co*-AAM)) for immobilization of myoglobin on MWCNTs. P(MAA-*co*-AAM) could be non-covalently attached to MWCNTs through hydrophobic interaction, resulting in the increase of solubility and stability of MWCNTs (Figure 10) [100]. In this work, myoglobin was immobilized onto the negative charged P(MAA-*co*-AAM)/MWCNTs surface through electrostatic interaction. Liu et al. developed a flow injection amperometric CNT based glucose biosensor using cationic polydiallyldimethylammonium chloride (PDDA). The CNT was firstly treated to achieve negative charged surface. Two layers of PDDA were then applied on CNT surface with layer-by-layer process. Glucose oxidase (GO*_x_*) was immobilized onto CNT surface by alternative assemble with PDDA layer and GO*_x_* layer. The sandwich structure formed by ionic interaction provided a favorable condition to keep bioactivity of GO*_x_*. The PDDA/GO*_x_*/PDDA/CNT/GC biosensor displayed excellent sensitivity towards H_2_O_2_ with a detection limit of 7 μM [105].

Polyethylenimine (PEI) is a cationic polymer with high density of amines in its structure, which makes it a good candidate for biomolecule immobilization through electrostatic interactions [101]. Ivnitski et al. developed a glucose biosensor using a MWCNT-modified electrode. A PEI- functionalized MWCNT was used as binder for immobilization of negatively charged glucose oxidase [102]. Liu et al. also applied PEI functionalization for immobilization of DNA onto MWCNT surface. PEI was grafted onto MWCNT in the presence of amine functionalized MWCNT by cationic polymerization. The resulting PEI-MWCNT demonstrated good stability and biocompatibility. DNA was securely immobilized onto PEI-MWCNT surface through electrostatic interaction. The amount of PEI on MWCNT surface was characterized by thermogravimetric analysis (TGA) [103]. Viswanathan et al. developed an electrochemical immunosensor for the detection of carcinoembryonic antigen (CEA) saliva and serum. Monoclonal anti-CEA antibodies (αCEA) were immobilized onto polyethyleneimine-wrapped MWCNT. The positively charged imine polymer chain attracted the molecules onto electrode surface, which facilitated electron transfer and resulting in higher peak current [38,104].

#### 2.2.4. CH-Π Interactions

**Figure 11:**
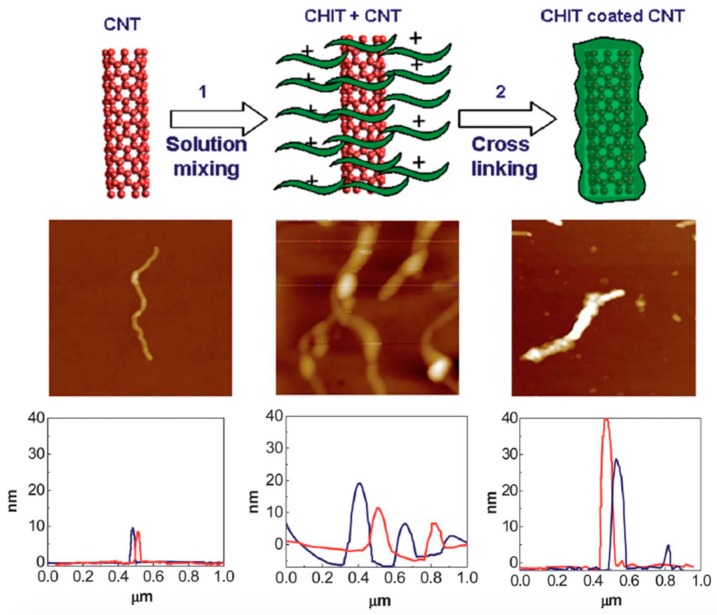
Schematic representations of Chit-f-CNT preparation (**top**), corresponding AFM height images (**middle**) and profile measurements (**bottom**) for pure CNT (**left**), Chit non-covalent functionalized CNT (**center**) and Chit covalently linked CNT (**right**).

Another approach of non-covalent functionalization of CNTs is to use amphiphilic molecules, which could interact with both hydrophilic and hydrophobic specie, where the hydrophobic side would interact with the nanotube surface [18]. Researchers have investigated that natural polysaccharides, such as amylose, alginate sodium, and chitosan could wrap onto CNTs sidewalls through non-covalent interaction, endowing different surface properties for CNT composites to interact with biological systems [108]. Kumar et al. studied the three electrode surfaces with deposition of CNTs, chitosan covalently functionalized CNTs, and chitosan non-covalently functionalized CNTs. As shown in Figure 11, the diameter of CNTs increases from 10 to 20 nm to up to 70–100 nm (non-covalent) and then to 50–80 nm (covalent). The authors concluded that chitosan have packed more densely when covalently linked to CNTs surface than in the non-covalent functionalized CNTs. Atomic force microscopy (AFM) could be used to characterized the effectiveness of coating of macromolecules [109].

Cyclodextrins (CD), for example, are groups of cyclic oligosaccharides with hydrophobic interior environments in their cavity structure [110]. Yang et al. successfully immobilized glucose oxidase on the film of polycyclodextrin and CNT, where the enzyme maintained its bioactivity due to the biocompatibility of cyclodextrin. The modified electrode was prepared by mixing CNT with solution of cyclodextrin and cyclodextrin prepolymer (pre-CD). CD can be adsorbed on CNT via Van der Waals forces along CNT structure. The synthesized composite was further used for immobilization of glucose oxidase. The electrochemical measurement showed that the CD/CNT film maintained the electrocatalytic activity of CNT and showed high sensitivity to glucose with a detection limit of 3.5 μM under pH 5.6–7.8 [111]. Although CH-π interaction provides only a tenth of the strength provided by hydrogen bonds, long molecules containing CH linkages could be sufficiently adsorbed onto CNTs and form stable complex under certain conditions. The CH-π multiple interactions significantly influence several chemical and biological phenomena. Baskaran et al. explored the effect of CH-π interaction in several polymer-MWCNT composites, such as polybutadiene-MWCNT, polystyrene-MWCNT and poly(methyl mechacrylate) (PMMA)-MWCNT, etc. The interaction was confirmed by IR and Raman frequencies of the composites [112]. Su et al. studied the controllable adsorption of MWCNTs onto C_18_H_37_SH deposited Au electrode via CH-π interaction. The surface coverage of MWCNTs could be easily controlled by adjusting the immersion time of MWCNTs onto C_18_H_37_SH. The modified electrode was observed with reduced interfacial capacitance compared with direct adsorption of MWCNT onto electrode surface. This approach offers opportunities for modified electrode preparation in biosensor applications [113]. Similar strategy was adopted by Zhang et al. for detection of Ag^+^ in aqueous solution using gold electrode coated with self-assembled 1-dodecanethiol (SC_12_H_25_). SWCNTs could attach to alkane groups through hydrophobic interaction. The detection limit of the biosensor is 1.5 nM which is much better than the required concentration limit from US Environmental Protection Agency for drinking water (0.46 μM) [114].

#### 2.2.5. Non-Covalent Functionalization without Coupling Agent

In addition to the functionalization methods introduced above, non-covalent functionalization of CNTs could also be achieved without coupling agent. Direct physical adsorption is simple and easy to establish by incubating the support materials in a solution containing the biomolecules such as enzymes [115]. Karajanagi et al. examined structure and function of enzyme soybean peroxidase (SBP), and α-chymotrypsin (CT), adsorbed onto SWCNT. CT only retained about 1% of its original activity upon adsorption [116]. This study was the first in-depth investigation of interaction between protein structure and carbon nanotubes, which is key criteria for successful biosensor development. The interaction between CNTs and enzymes could influence the enzyme secondary structure and function. Although many enzyme-based biosensors utilizing carbon nanotubes were developed using physical adsorption, the physical bonding is generally too weak to keep the biomolecule immobilized on CNTs surface and is prone to enzyme leakage from the matrix [117,118,119,120,121].

In all, non-covalent immobilization of CNTs could help improve the biocompatibility of CNTs for a variety of different applications, such as DNA assays, enzymatic biosensors, or bacterial detection, etc. The major advantage of non-covalent immobilization of CNTs using aromatic molecules or polymers is resulting increase in CNT solubility, while retaining the conjugate structure of CNTs which plays an important role in the electrochemical activity of the electrodes. The functionalization processes are generally straightforward and easy to conduct using various coupling agents as discussed previously. A variety of different characterization techniques could be applied to characterize the effectiveness of the functionalization, such as UV-Vis spectroscopy, Raman spectroscopy, AFM, SEM, and TEM, etc. However, in non-covalent immobilization of CNTs, the bonding between the coupling agent and CNTs is relatively weaker compared to covalent linkage, which may affect the stability of the established systems. This trade-off must be considered in determining the choice of functionalization methods of CNTs for specific applications.

## 3. Applications of Non-Covalent Functionalization of CNT Using Different Bio-Recognition Elements for Electrochemical Biosensors

Carbon nanotubes have been extensively studied and used as transducers for electrode construction in electrochemical biosensors, due to their high electrical conductivity, biocompatibility, and high surface area. Carbon nanotubes transducers are used for the detection of molecules of biological significance through surface modification with analyte-specific molecules (recognition element), such as protein, enzyme, antibody, virus and DNA, etc. The recognition element is able to bind targets specifically and cause physical/chemical change, resulting in shifts in electronic signals. Herein, we discuss the examples of carbon nanotube based electrochemical biosensors that were developed by using non-covalent functionalization of CNT with recognition molecule.

### 3.1. Proteins

Many proteins have been utilized in biosensor applications due to their excellent biocompatibility and abundant surface groups. The employment of proteins has not only been used as recognition molecule in detection of target molecules, but also as amphiphilic biomolecule for CNT surface functionalization. Chen et al. reported for the first time, a simple and general approach to non-covalently functionalize side-walled carbon nanotubes using PBSE. The functionalized SWCNT was then used for immobilization of ferritin and streptavidin, which were successfully observed under TEM. This approach could be extended beyond biomolecules, and could be utilized in various applications, such as polymerizable molecules or small molecules with desired properties [50].

Zhan et al. reported a facile method of myoglobin immobilization on MWCNTs using poly(methacrylic acid-*co*-acrylamide) (P(MAA-*co*-AAM)), for H_2_O_2_ detection. The resulting P(MAA-*co*-AAM)/MWCNTs composites provide active ligands like carboxyl and amine groups for protein/enzyme immobilization and facilitate electron transfer during electrochemical reaction. The fabricated electrochemical biosensor displayed a detection limit as low as 0.76 μM towards H_2_O_2_ under optimal experimental conditions [100]. Another class of biomolecules called hydrophobins, which are small proteins that have both hydrophobic and hydrophilic parts which realize easy self-assembling at various interfaces. In contrast to regular surfactant, the surface activity of hydrophobin depends on the change of molecule conformation during self-assembly rather than on a diffusion-limited adsorption to the interface. Wang et al. demonstrated a simple way of non-covalent functionalization of MWCNTs with hydrophobin (HFBI) through hydrophobic interaction to increase the solubility of MWCNT. The performance of the hybrid structure was evaluated using an amperometric biosensor for detection of glucose. The resulting nanocomposite exhibit excellent electron transfer ability and electrocatalytic activity with low detection limit of 8.2 μM and a sensitivity of 116 μA·mM^−1^·cm^−2^ [122].

### 3.2. Enzymes

The nanowire structure of CNTs enables the approach to the active center of redox enzyme, resulting in fast and efficient electron transfer. In some cases, enzyme spontaneously physisorbs onto CNTs surface based on hydrophobic interaction and achieves direct electron transfer [93]. Appropriate functionalization of CNTs helps anchor biomolecules with its active site close enough to electrode surface or with redox active species to establish mediated electron transfer. Besteman et al. demonstrated the use of semiconducting SWCNTs via the same linking molecule PBSE reported previously for detection of glucose. The sensor was also found to act as a pH sensor with reversible changes in conductance upon changes in pH [31].

Bourourou et al. reported a non-covalently functionalized MWCNT with anthraquinone derivatives bearing pyrene groups to enhance the direct electron transfer between electrode and T1 copper site of laccase. The enzyme was immobilized with high stability based on the hydrophobic interaction between pyrene-MWCNT sidewalls and anthraquinone- laccase [54]. Goff et al. reported a synthesized complex tris[4,4-bis(4-pyren-1-ylbutyloxy)bipyridinyl] iron (II) complex, containing six pyrene groups, that help the functionalization of MWCNTs as well as the immobilization of β-cyclodextrin tagged glucose oxidase for detection of glucose. The formation of pyrene/β-cyclodextrin linkage during enzyme immobilization provides maximum quantity of immobilized enzyme onto electrode surface while ensuring the diffusion of H_2_O_2_. The limit of detection for glucose was 1.5 μM·L^−1^ and the sensitivity was determined to be 13.5 mA·L·M^−1^·cm^−2^ [123].

**Figure 12:**
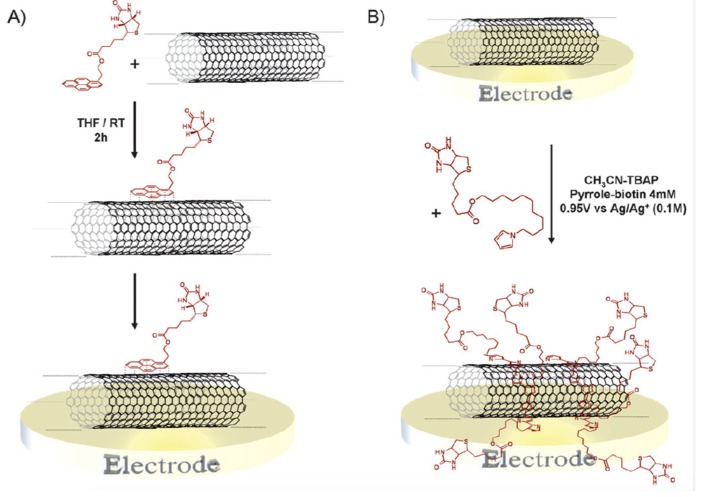
Biotin functionalization on nanotubes using (**A**) π-π stacking interaction between pyrene-biotin derivatives and the nanotube side walls; (**B**) Electropolymerization of the pyrrole-biotin monomer on the SWCNT [124].

Haddad et al. studied the non-covalent functionalization of SWCNTs with biotin for the construction of glucose biosensors (Figure 12). The functionalization of SWCNTs was achieved either by electropolymerization of biotinylated pyrrole derivatives or formation of π-π stacking using biotinylated pyrene derivatives. The resulting SWCNTs were used for successive immobilization of avidin and biotin labelled glucose oxidase through avidin-biotin affinity interaction. A reduced H_2_O_2_ diffusivity was observed for electropolymerized film functionalized biosensor, whereas an optimal permeability of H_2_O_2_ was observed for the pyrene-functionalized CNTs. The highest sensitivity was found to be 5.2 mA·M^−1^·cm^−2^ with a maximum current density of 33.5 μA·cm^−2^ for 60 μL of the carbon nanotube deposits [124].

A similar glucose biosensor was developed using poly[3-(3-N,N-diethylaminopropoxy) thiophene] (PDAOT), a conjugate polymer. Glucose oxidase was entrapped within PDAOT-SWCNT film. The fabricated Au/PDAOT-SWCNT/GO*_x_* biosensor exhibited fast current response with detection limit of 5 μM and a sensitivity of 700 ± 26 μA·mM^−1^·cm^−2^ [99]. Zhu et al. reported a bi-enzyme amperometric biosensor for selective and sensitive detection of glucose. SWCNT, non-covalently functionalized with electropolymerized pyrrole, was used as the substrate for enzyme immobilization. The bi-enzyme system, consisting of glucose oxidase (GO*_x_*) and horseradish peroxidase (HRP), was entrapped in the electropolymerized pyrrole film. Direct electron communication was established by SWCNT between HRP and electrode. The detection limit of the biosensor was 0.50 ± 0.14 μM (S/N = 3) and the sensitivity was found to be 430 ± 13.4 μA·mM^−1^·cm^−2^ [96].

**Figure 13:**
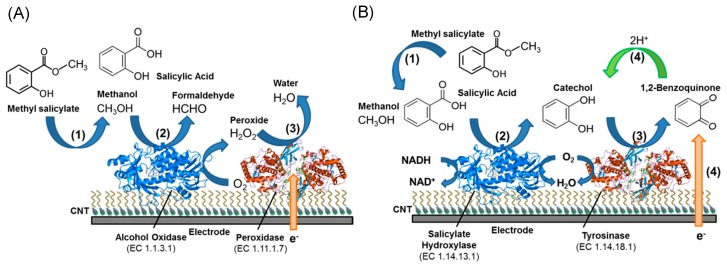
Schematic illustration of methyl salicylate detection using two types of bi-enzyme based carbon nanotube/PBSE modified biosensor with (**A**) Alcohol oxidase and horseradish peroxidase and (**B**) salicylate hydroxylase and tyrosinase [125,126].

Silveira et al. reported amperometric nitrite biosensor based on the heterogeneous electron exchange between nitrite reductase (ccNiR) and SWCNT modified electrode. SWCNT was modified with polypyrole and the carbon coating offered enlarged surface area and higher active sites for immobilization of ccNiR. At optimal condition, the biosensor sensitivity towards nitrite was 2.4 ± 0.1 A·L·M^−1^·cm^−2^. The prepared SWCNT/ccNiR with additional polymer layer preserved high stability for about 90% after 20 days [95]. Periasamy et al. developed an amperometric glucose biosensor using gelatin-MWCNTs modified glassy-carbon electrode. Non-polar amino acid chain of gelatin was immobilized on the sidewall of MWCNTs through hydrophobic interaction. GO*_x_* was further immobilized onto gelatin-MWCNTs through glutaraldehyde chemistry. A rapid electron transfer was observed between GO*_x_* and the glassy-carbon electrode with a sensitivity of 2.47 μA·mM^−1^·cm^−2^. In addition to the monoenzymatic biosensors introduced above, our recent researches developed bi-enzymatic biosensors for the detection of methyl salicylate (Figure 13). Salicylate hydroxylase and tyrosinase were immobilized onto MWCNTs using PBSE as cross-linker, and the sensitivity and limit of detection were 30.6 µA^−1^·cm^−2^·µM^−1^ and 13 nM [125,126].

### 3.3. Antibodies

Antibody-antigen interaction is the most common affinity interaction in biosensor applications. Other types of affinity interactions include biotin-streptavidin interaction, avidin-biotin and host-guest complex (adamantane-cyclodextrin) [61,98]. The paratope of antibody could interact with the epitope of antigen by spatial complementarity, which could be utilized in the development of immunosensors by incorporating antibody or antigen with different transducers. CNTs are also increasingly being used in immunosensor fabrication. CNTs could function as transducers, carriers or labels of immunoassays since they could transfer large amount of electroactive species to amplify the electrochemical signals as well as stabilizing the bioactive species [127]. Tam et al. investigated immunosensor using CNT/antibody doped polypyrrole (Ppy). The CNT/Ppy/Antibody composite was synthesized through electrochemical deposition. Pyrrole polymer and antibody goat-IgGs were slowly polymerized onto CNT coated electrode. The detection limit of the immunosensor was as low as 0.05 μg·mL^−1^ with fast response time [97]. Chen et al. proposed an approach for antibody immobilization using polyethylene oxide functionalized SWCNT. The selective antibody was then conjugated with the functionalized SWCNT for detection of human autoimmune diseases [47]. Similarly, Sánchez et al. developed electrochemical immunosensor using a nanotube/ polysulfone/RIgG composite. Screen printed electrode modified with polysulfone functionalized CNTs was used as working electrode. Rabbit IgG was then used as the model antibody labeled with horseradish peroxidase (HRP), and it was incorporated into polysulfone membrane/MWCNT hybrid and incubated with anti-RIgG-HRP. Direct electrochemical response of HRP was achieved by addition of H_2_O_2_ in solution. The detection limit was determined to be 1.66 μg·mL^−1^ and the linear range of anti-RIgG was from 2 to 5 μg/mL [128].

Cui et al. investigated an electrochemical immunosensor using gold nanoparticles (GNPs) and CNTs with HRP for detection of human IgG as model protein using glass carbon electrode. Upon absorption of GNPs onto CNTs surface, antibody was immobilized onto GNPs due to the strong interaction between GNPs and mercapto or primary amine groups in biomolecules. A linear response range was achieved between 0.125 and 80 ng/mL with a detection limit of 40 pg/mL. The developed method offers enhanced performance and could be extended to other protein detection schemes [129].

### 3.4. Viruses

**Figure 14:**
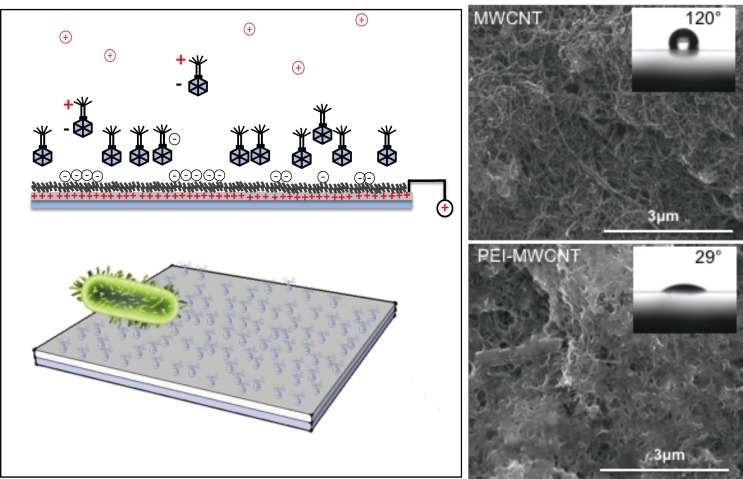
Schematic of the charge-directed orientation and immobilization of bacteriophages onto PEI-functionalized CNT on electrode surface, and the SEM images of CNT before and after PEI modification [130].

Viruses have also been used as recognition elements integrated with CNT for detection of the target cells. In our previous publications, we have reported a phage-based electrochemical biosensor for detection of *Escherichia coli* using polyethylenimine (PEI)-functionalized carbon nanotube as shown in Figure 14 [130,131]. An electrostatic interaction can be achieved by using positively charged polyethylenimine-functionalized carbon nanotube (PEI-CNT) as supporting material, which enables the positioning of phage particle in the right orientation on the electrode. The PEI-CNT showed increased surface energy and made the surface more hydrophilic. A reliable and selective detection of *E. coli* was achieved using the T2 phage-based biosensor with a linear response range between 10^3^ CFU·mL^−1^ and 10^7^ CFU·mL^−1^ and a detection limit of 10^3^ CFU·mL^−1^. The interaction between virus and carbon nanotubes have been studied via phage display technology. Yu et al. applied phage display technology for selecting phage peptides having affinity towards different chiral structures of SWCNTs. A virus-based biological template, M13 bacteriophage, for selecting single-walled CNTs’ chirality was developed and reported in 2011, based on the premise that interaction between the peptide and CNTs is sensitive to amino acid sequences [132].

### 3.5. DNA

**Figure 15:**
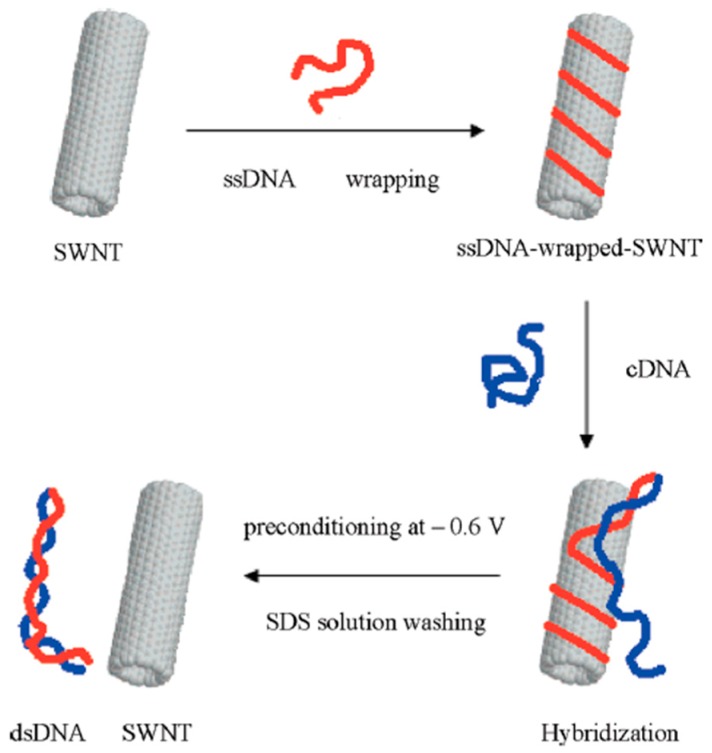
Schematic illustration of the interaction between SWCNT and DNA [136].

Biosensors for detection of DNA are rapidly developing as an alternative to the classical genetic assays. For the detection of target DNA sequence, DNA biosensors are designed with a transducer combined with DNA, which is called the probe, acting as the recognition element. A variety of DNA could interact with CNTs including single stranded DNA (ssDNA) and short double stranded RNA (dsRNA), while there is no evidence for double stranded DNA (dsDNA) wrapping on SWCNTs [133]. It was reported that ssDNA either small oligonucleotides consisting of tens of bases or complete sequence, can wrap around SWCNTs [134,135]. Zhang et al. fabricated a DNA hybridization biosensor based on interaction between ssDNA and SWCNT. SWCNT array electrode was prepared by wrapping with ssDNA via hydrophobic interaction without any bridging agent as shown in Figure 15. Differential pulse voltammetry (DPV) response was recorded during DNA hybridization process. Such biosensor was reported to be reusable for 3000 times to detect different types of DNA with a good linear response between 40 and 110 nM with a detection limit of 20 nM [136]. Aravind et al. reported a hybrid nanocomposite consisting of Pt decorated MWCNT immobilized with ssDNA for selective detection of dopamine. The established hybrid nanocomposite biosensor exhibits a linearity response up to ∼315 μM, with a detection limit 0.8 μM towards dopamine [33]. Moreover, DNA can be removed from SWCNTs via hybridization, in order to detect target DNA sequence. One of the most recent DNA sensor applications is to detect genetically modified organisms (GMO) in food. DNA recognition is used to hybridize the target DNA sequence with GMO-specific probes that are immobilized on the surface of the sensor [137].

The use of non-covalent functionalized CNT renders the cationic groups available for negatively charged DNA binding via ionic interaction. Sanz et al. reported a bilayer approach for non-covalent functionalization of SWCNT using RNA-wrapping method. However, RNA-wrapping conferred negative charges on CNT, which made it unsuitable for DNA binding. By using cationic polymer as bridging molecule, such as polyethyleneimine (PEI), poly(Lys:Phe, 1:1) and polylysine, CNT surface was positively charged and ready for DNA binding [104].

Ensafi et al. developed a sensitive MWCNT-based biosensor for detection of amitrole in water and soil sample. The working electrode was modified with MWCNT and positively charged chitosan with negatively charged DNA immobilized on the electrode surface through electrostatic interaction. It was claimed that amitrole interacted with dsDNA mainly through an intercalation mode, which increased the differential pulse oxidation wave of amitrole 20 times higher than that of a bare electrode. The sensor demonstrated satisfactory selectivity and sensitivity with a detection limit of 0.017 ng·mL^−1^ [138].

## 4. Conclusions and Outlook

Carbon nanotubes possess unique properties that are different from conventional materials, such as large surface area, excellent electronic properties and high conductivity, which make them suitable for biosensor applications. This article reviews different strategies for non-covalent functionalization of CNT. Unlike covalent functionalization, non-covalent functionalization of CNT helps retain pristine mechanical and electronic properties of CNTs and preserve the intrinsic properties with desirable functionality. Non-covalent functionalization of CNT for biosensors provides an ideal strategy for biomolecule immobilization with high biocompatibility. Although non-covalent functionalization of CNTs using aromatic molecules has attracted attention recently, only few comprehensive studies have been carried out so far on the effect of interaction mechanisms and CNT properties (length, curvature, chirality, etc.) on the effectiveness of non-covalent functionalization. This versatile and multifunctional CNTs nanostructures could be potentially employed for diagnostic and therapeutic applications.

## Figures and Tables

**Table 1:** List of applications that use covalent functionalization for biomolecule immobilization on carbon nanotubes.

Crosslinker	Biomolecule	Type of CNT	Application	Reference
EDC/PLL *	HRP	COOH-SWCNT	H_2_O_2_ sensing	[35]
EDC-NHS	GO*_x_* and HRP	COOH-CNT	Glucose biosensor	[36]
EDC-NHS	Antibody	COOH-MWCNT	Aflatoxin detection	[37]
GA	Antibody	PEI-MWCNT	Carcinoembryonic antigen detection	[38]
GA	GO*_x_*	Gelatin-MWCNT	Glucose biosensor	[39]

* EDC: N-ethyl-N’-(3-dimethylaminopropyl) carbodiimide hydrochloride; NHS: N-hydroxy-succinimide; PLL: poly-l-lysine; GO*_x_*: glucose oxidase; HRP: horseradish peroxidase.

**Table 2:** List of aromatic compounds used for non-covalent functionalization of carbon nanotubes.

Compound Name	Structure	CNT Structure	Application	References
1-Pyrenebutanoic acid, succinimidyl ester (PBSE)	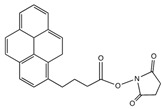	SWCNT MWCNT	Bioelectrocatalysis of oxygen	[27,45,50,51,52,53,54]
1-(2-Anthraquinonylamino-methyl)pyrene	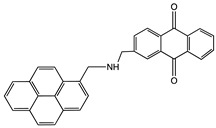	MWCNT	Bioelectrocatalysis of oxygen	[54]
[bis(2-anthraquinonyl)-aminomethyl]pyrene	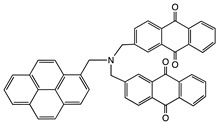	MWCNT	Bioelectrocatalysis of oxygen	[54]
5,7,12,14-Tetramethyl-dibenzo-1,4,8,11-tetraazacyclotetradeca-3,5,7,10,-12,14-hexaene (TMTAA)	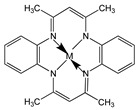	SWCNT	Not available	[60]
Adamantane-pyrene	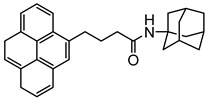	SWCNT	Glucose biosensor	[61]
Biotin–pyrene	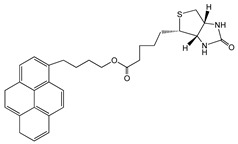	SWCNT	Glucose biosensor	[61]
Nitrilotriacetic acid (NTA)–pyrene	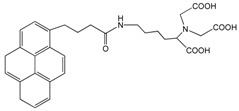	SWCNT	Glucose biosensor	[61]
Ferrocene	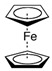	SWCNT	Glucose biosensor/ L -glutamate detection	[59,62,63]
Anthracene	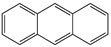	SWCNT	Bioelectrocatalysis of oxygen	[64,65]
Anthrarobin	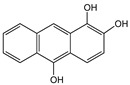	SWCNT	Not available	[65]
9,10-Dibromoanthracene	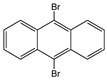	SWCNT	Not available	[65]
9,10-Anthracene-dicarbonitrile	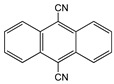	SWCNT	Not available	[65]
9-Anthracenemethanol	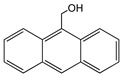	SWCNT	Not available	[65]
Thionine	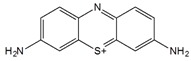	MWCNT	Glucose and uric acid biosensors/ Hydrogen peroxide detection	[66,67,68,69,70,71]
Methylene blue (MB)	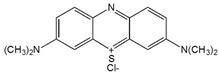	SWCNT	Not available	[72,73]
Naphthalen-1-methyl- phosphonic acid (NYPA)	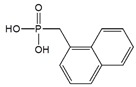	SWCNT	Hydrogen peroxide detection	[74]
1,10-Phenanthroline-5,6-dione (PD)	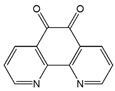	MWCNT	Ethanol biosensor	[75]
Triphenylene (TP)	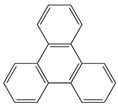	MWCNT	TNT detection	[76]
Porphyrin	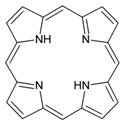	SWCNT	Not available	[77]
Hydroxyferriproto- porphyrin (hematin)	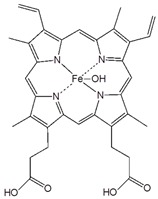	SWCNT	Trichloroacetic acid biosensor	[78]

**Table 3:** List of polymers used for non-covalent functionalization of carbon nanotubes.

Compound Name	Structure	CNT Structure	Application	References
Polypyrrole (PPy)	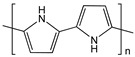	SWCNT	Nitrite detection/ anti-goat IgGs detection	[94,95,97]
Adamantane-pyrrole	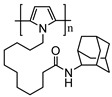	SWCNT	Glucose biosensor	[98]
Poly[3-(3-N,N-diethyl-aminopropoxy)thio-phene] (PDAOT)	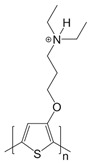	SWCNT	Glucose biosensor	[99]
Poly(methacrylic acid-*co*-acrylamide) (P(MAA-*co*-AAM))	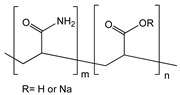	MWCNT	Hydrogen peroxide detection	[100]
Polyaniline (PANI)	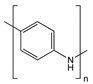	MWCNT	Carbamate pesticides detection	[93]
Glycodendrimer	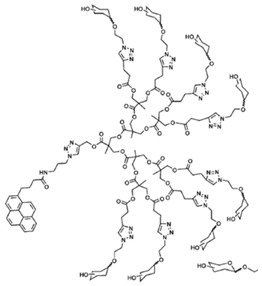	SWCNT	Metal detection	[27,52]
Polyethyleneimine	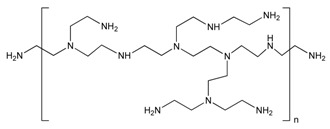	MWCNT	Glucose biosensor/DNA delivery/ Carcinoembryonic antigen detection	[38,101,102,103,104]
Poly diallyl dimethyl ammonium chloride (PDDA)	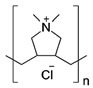	SWCNT	Glucose biosensor	[105]
